# Regression of Liver Steatosis Following Phosphatidylcholine Administration: A Review of Molecular and Metabolic Pathways Involved

**DOI:** 10.3389/fphar.2022.797923

**Published:** 2022-03-10

**Authors:** D. Osipova, K. Kokoreva, L. Lazebnik, E. Golovanova, Ch. Pavlov, A. Dukhanin, S. Orlova, K. Starostin

**Affiliations:** ^1^ Research Centre for Medical Genetics, Moscow, Russia; ^2^ Institute of Pediatric Endocrinology, Endocrinology Research Centre, Moscow, Russia; ^3^ A. I. Evdokimov Moscow State University of Medicine and Dentistry, Ministry of Health of Russia, Moscow, Russia; ^4^ I. M. Sechenov First Moscow State Medical University, Ministry of Health of Russia, Moscow, Russia; ^5^ Molecular Pharmacology and Radiology Department, Russian National Research Medical University, Moscow, Russia; ^6^ Department of Dietetics and Clinical Nutrition of Continuing Medical Education, Medical Institute, RUDN University, Moscow, Russia; ^7^ Science Hub, Sanofi, Moscow, Russia

**Keywords:** liver steatosis, essential phospholipids, mode of action, pharmacodynamics, phosphatydilcholine, nonalcoholic fatty liver, review

## Abstract

Liver steatosis is a key pathology in non-alcoholic or metabolic associated fatty liver disease. Though largely ignored for decades it is currently becoming the focus of research in hepatology. It is important to consider its origin and current opportunities in terms of pharmacotherapy. Essential phospholipids (EPLs) rich in phosphatidylcholine (PCH) is a widely used treatment option for fatty liver disease, and there is a solid amount of consistent clinical evidence for the regression of steatosis after treatment with EPLs. As knowledge of PCH (a key component of EPLs) pharmacodynamics and mode of action driving this widely observed clinical effect is currently insufficient, we aimed to explore the potential molecular and metabolic pathways involved in the positive effects of PCH on steatosis regression.

## Introduction

Non-alcoholic fatty liver disease (NAFLD) is the leading cause of chronic liver disease (CLD), which puts it among the top global health priorities. NAFLD prevalence is increasing dramatically every year. NAFLD was responsible for 46.8% of all chronic liver disease cases in 1994 and 75.1% in 2008 (Younossi et al., 2011). Nowadays it is the second most frequent indication for liver transplantation in the United States (Holmer et al., 2017). NAFLD is expected to be the most common cause of liver transplantation by 2030 (Byrne and Targher, 2015; Tana et al., 2019).

Numerous studies aimed to find the best treatment for NAFLD/NASH (non-alcoholic steatohepatitis). Dozens of clinical trials and studies have been performed recently to assess the efficacy and safety of different candidate molecules. Unfortunately, most of these molecules have fallen short of expectations (cenicriviroc in the CENTAUR study (Friedman et al., 2018), obeticholic acid in the REGENERATE study (Ratziu et al., 2019; Younossi et al., 2019), or elafibranor in the RESOLVE-IT study (NCT02704403)). Vitamin E in high dosage (800 IU) showed some effect in NASH patients, but there was a relapse in inflammation markers after the end of treatment, and authors raised concerns about vitamin E safety profile if taken constantly in such a dosage (Sanyal et al., 2010; Lavine et al., 2011). Most of these new molecules were used to treat NASH and/or advanced fibrosis, but not the steatosis stage of the disease, for which lifestyle modification has been considered so far as the only treatment option. At the same time, less than 2% of obese patients have reached normal weight in a large real-world data setting (Fildes et al., 2015). So, lifestyle modification, being considered as a key NAFLD treatment (Francque and Vonghia, 2019), is largely useless for 98% of patients (Fildes et al., 2015). Moreover, the concept of steatosis as a physiologically adaptive mechanism is outdated. Nowadays it is challenged, and steatosis is becoming the focus of clinical and scientific interest as a condition increasing cardiovascular risks and mortality and, therefore, requiring pharmacotherapy (Zhou et al., 2012; Adams and Ratziu, 2015; Nassir et al., 2015; Francque and Vonghia, 2019). In this respect, it is also worth mentioning that NAFLD is reconsidered as liver steatosis associated with metabolic disorders and thus may be renamed as MAFLD, i.e., metabolic (disorders) associated fatty liver disease. MAFLD concept cancels NAFLD/NASH dichotomy, with liver steatosis as a key diagnostic criterion along with various metabolic disorders.

Putting this together we suggest that steatosis should be considered not only as a key diagnostic criterion but a target for pharmacotherapy to be combined with lifestyle modification. Simple steatosis is known to be fully reversible and, thus, should be treated before steatohepatitis develops (Choudhary et al., 2015).

Among the existing pharmacotherapeutic options, essential phospholipids (EPLs) containing 72–96% (3-sn-phosphatidyl)choline are of interest as recent randomized controlled trials and meta-analyses showed regression of steatosis associated with EPLs treatment (Gundermann et al., 2016; Popovic and Dajani, 2020). A large observational study showed a similar effect in a real-world setting (Maev et al., 2020b). At the same time, despite abundant evidence of the clinical effect of EPLs, their mode of action is still poorly understood. Our review is aimed to analyze the key potential molecular pathways involved in the clinical effect of PCH, the main component of EPLs.

### Essential Phospholipids Source and Chemical Profile

Generally, EPLs are natural phospholipids that can be plant-derived (e.g., from soybeans, rape (canola) seed, wheat germ, sunflower, or flaxseed) or animal-derived (e.g., from egg yolk, milk, or krill). Phospholipids are called essential since they constitute structural and functional components of all cell membranes and, therefore, endogenous substances. The phospholipid content of membranes and the distribution of fatty acid residues vary within a cell and between cell types (Pepeu et al., 1990). The distribution of different types of phospholipids in cells and organs is not yet fully understood, but the interaction of various phospholipids and other membrane components seems to have an important role in signal transduction cascades (Van Hoogevest and Wendel, 2014).

The phospholipid and fatty acid profiles of the EPLs depend on the raw material sources, but for EPLs generally used in liver diseases, most sources use the following well-recognized definition of EPLs. EPLs are a highly purified extract of the semen of soybeans with standardized contents of 72–96% (3-sn-phosphatidyl)choline (PCH) (Gundermann et al., 2011). In most studies in humans EPLs with 76% of PCH were administered (Popovic and Dajani, 2020). The chemical profile of soybean lecithin with 73–79% of PCH is provided in Table 1. The process of obtaining EPLs from soybean is well-described elsewhere (Van Hoogevest and Wendel, 2014). The quantitatively and qualitatively dominating molecule is 1,2-Dilinoleoylphosphatidylcholine (DLPC), representing up to 52% of the administered phosphatidylcholine molecules. This high level of DLPC is the primary difference between EPLs and typical unprocessed (natural) phospholipids (e.g., triple lecithin, raw lecithin, and egg lecithin), as well as dietary and endogenous phosphatidylcholines (Gundermann et al., 2011).

**TABLE 1:** Phospholipid composition of the soybean lecithin extract and chromatography fraction with PC content of 73–79% (Van Hoogevest and Wendel, 2014).

Component (% w/w)	Fraction with PCH 73–79%
PCH	79
PE	3.3
PI	0.2
PA	1.6
LPC	6.1
*N*-Acyl-PE	1.7

PCH, phosphatidylcholine; PE, phosphatidylethanolamine; PI, phosphatidylinositol; PA, phosphatidic acid; LPC, lysophosphatidylcholine; *N*-Acyl-PE, phosphatidylethanolamine.

Since PCH is a dominating component of EPLs, our review is focused on the analysis of the role of PCH in liver steatosis regression. First of all, we took into consideration liver steatosis pathophysiology and fatty liver modeling approaches to provide an overview of involved metabolic and molecular pathways. Second, we considered it in terms of potential PCH influence on the key processes involved in liver steatosis pathophysiology.

## Liver Steatosis Pathophysiology

Non-alcoholic fatty liver disease (NAFLD), or MAFLD, as it has been renamed in 2020, is a clinical diagnosis involving the presence of at least 5% hepatocytes with lipid droplets observed by microscopy of biopsy material or fatty infiltration revealed by imaging tests and excluding all secondary causes of excessive accumulation of triglycerides (TG) in the liver (Carr et al., 2016).

So, the key pathology is liver steatosis. It is of great interest what the true cause is for *de novo* lipogenesis in hepatocytes. Several models explaining the origins of steatosis exist (Kim et al., 2000; Kotani et al., 2004; Petersen et al., 2007). Of these models, the following are considered most reliable and useful in terms of further research:

1) Metabolic pathway of liver steatosis.

Insulin resistance of peripheral tissues → transient hyperglycemia → glucose uptake by the liver →liponeogenesis → accumulation of TG in the liver (Kim et al., 2000; Kotani et al., 2004; Petersen et al., 2007; Liu et al., 2010). In this case, prediabetes is considered the leading cause of NAFLD.

2) Alimentary pathway of liver steatosis.

Kcal overload → lipid transformation and TG accumulation in the liver → insulin resistance of liver cells (Samuel et al., 2004)→systemic insulin resistance (Cai et al., 2005). In this case, NAFLD may be the primary metabolic disorder representing hepatic manifestations of metabolic syndrome leading to prediabetes.

3) Mixed pathway of liver steatosis.

In real-life clinical practice, we believe that both kcal overload and insulin resistance may develop and exist in parallel. In this case, both prediabetes and NAFLD clinical manifestations would be present at the same time. Depending on the dominant pathway in a sample, NAFLD leading to prediabetes or prediabetes leading to NAFLD is observed in different studies which reflects the reciprocal interconnection (Xia et al., 2019).

**FIGURE 1:**
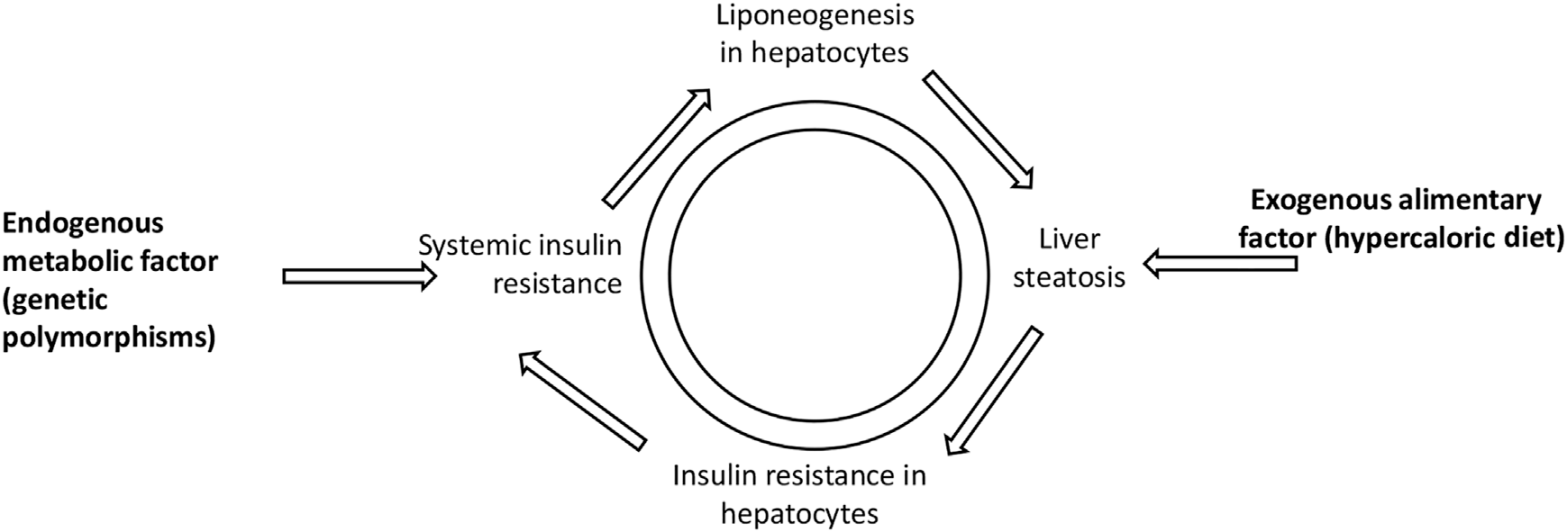
Vicious circle illustrating liver steatosis and prediabetes interconnection. Both exogenous alimentary factor (hypercaloric diet) and endogenous metabolic factor (genetic polymorphisms) may lead to systemic insulin resistance and liver steatosis.

The pathways of liver steatosis mentioned above may shed light on the correlation between liver steatosis and prediabetes (Chen et al., 2017). Such a vicious circle between fatty liver and prediabetes may be presented as follows (Figure 1).

Insulin resistance and liponeogenesis in hepatocyte molecular interconnection are described in detail elsewhere (Chen et al., 2017). For the following review of PCH mode of action, it is worth mentioning that hyperinsulinemia and hyperglycemia in NAFLD induce SREBP-1c (sterol regulatory element-binding protein-1c) and ChREBP (carbohydrate response element-binding protein), respectively, leading to lipogenic pathway activation causing conversion of excess glucose to fatty acids. Improved fatty acids synthesis results in increased levels of malonyl-CoA, which inhibits CPT-1 (carnitine palmitoyltransferase 1), the transporter of fatty acids to mitochondria. These events lead to a shift between free fatty acids beta-oxidation and *de novo* lipogenesis (Di Mauro et al., 2016).

Regardless of the exact pathway of TG accumulation in the liver, it may be characterized by impairment of the following processes:

1) Fatty acids and TG utilization in hepatocytes (lipolysis)
2) Fatty acids and TG de novo synthesis in hepatocytes (liponeogenesis)
3) Fatty acids and TG secretion or evacuation
4) Fatty acids and TG dietary intake

Therefore, we aimed to explore whether these processes were modified by PCH and may explain its clinical effect observed consistently in randomized controlled trials and observational clinical studies and proven in recent meta-analyses where EPLs were administered (Arvind et al., 2006; Sas et al., 2013; Dajani et al., 2015; Gundermann et al., 2016; Popovic and Dajani, 2020).

## Fatty Liver Modeling Studies

In 2005, Buang et al. conducted a perfect *in vivo* study in Sprague-Dawley rats fed a basic diet with TG (control group), TG and orotic acid (fatty liver model group), or orotic acid and phosphatidylcholine (PCH group) for 10 days (Buang et al., 2005). Liver weight was the same across groups at baseline, however, its increase differed at the end of the study in the fatty liver model group vs. the control group. Liver TG increased in the fatty liver model group, but not in the PCH group. Thus, the effect of PCH on steatosis in the liver was reproduced. Moreover, blood cholesterol and TG levels were minimal in the PCH + fatty liver group. Thus, lower levels of liver TG in the PCH group may be explained with fatty acids digestion, synthesis, or oxidation, rather than with excretion from hepatocytes. More importantly, Buang analyzed enzyme activity, which gave us another reason to continue the relevant literature search. The PCH group did show a change in the expression of enzymes involved in fatty acids synthesis and beta-oxidation (fatty acids catabolism). Particularly, in the PCH group, fatty acid synthase (FAS) and glucose-6-phosphate dehydrogenase (G6PDH) activity and malate dehydrogenase (ME) expression were decreased (involved in fatty acids synthesis), while carnitine palmitoyltransferase (CPT) expression increased (involved in beta-oxidation).

It is known that in the case of choline deficiency triglycerides cannot be removed effectively from the hepatocytes since choline is the precursor of PCH, and PCH is essential for very-low-density lipoproteins (VLDL) synthesis and excretion (Stephenson et al., 2018; Soret et al., 2020; Nababan et al., 2021). That is why fatty liver may be modeled with a choline-deficient diet (Kulinski et al., 2004; Testerink et al., 2009). In a study by Testerink, mutant Chinese hamster ovary cell line MT58 was used containing a thermosensitive mutation in phosphocholine cytidylyltransferase (CTP), the regulatory enzyme in the CDP-choline synthesis pathway. MT58 cells had a 50% decrease in PCH level within 24 h when cultured at the nonpermissive temperature, accompanied by an increase in the number of cytosolic lipid droplets (Testerink et al., 2009). In a study by Kulinski, mice were fed a choline-deficient diet (compared with a choline-supplemented diet) for 21 days, and liver triacylglycerol was increased, while plasma apolipoproteins (apo) ^100^B and B48 were decreased (Kulinski et al., 2004). There is also evidence that EPLs may influence the intestinal digestion of lipid molecules. For instance, Rampone et al. showed >50% suppression of cholesterol intestinal uptake when incubating it with different dosages of liver lecithin in everted rat gut sacs (Rampone, 1972). Everted rat gut sac model is a standard *in vitro* procedure to study drug absorption (Alam et al., 2012). We investigated these two directions of EPL mode of action as well to develop a unified pharmacodynamic picture.

## Lipolysis Stimulation

### PPAR as a Target Molecule for EPLs

Peroxisome proliferator-activated receptors (PPARs) are known to play a huge role in the regulation of energy homeostasis and metabolic functions, including lipid metabolism (Tyagi et al., 2011). These ligand-activated nuclear transcription factors belonging to the large nuclear receptor superfamily are expressed as three isoforms (PPARα, PPARβ/δ, and PPARγ). PPARα is expressed ubiquitously among body tissues with the highest concentration observed in the liver (Liss and Finck, 2017). Its fundamental function is to regulate fatty acids and triglycerides metabolism, beta-oxidation, and ketogenesis (Desvergne and Wahli, 1999). Because of that, PPARα is of high scientific interest in terms of lipid metabolism correction opportunities (Han et al., 2017a; Han et al., 2017b).

**FIGURE 2:**
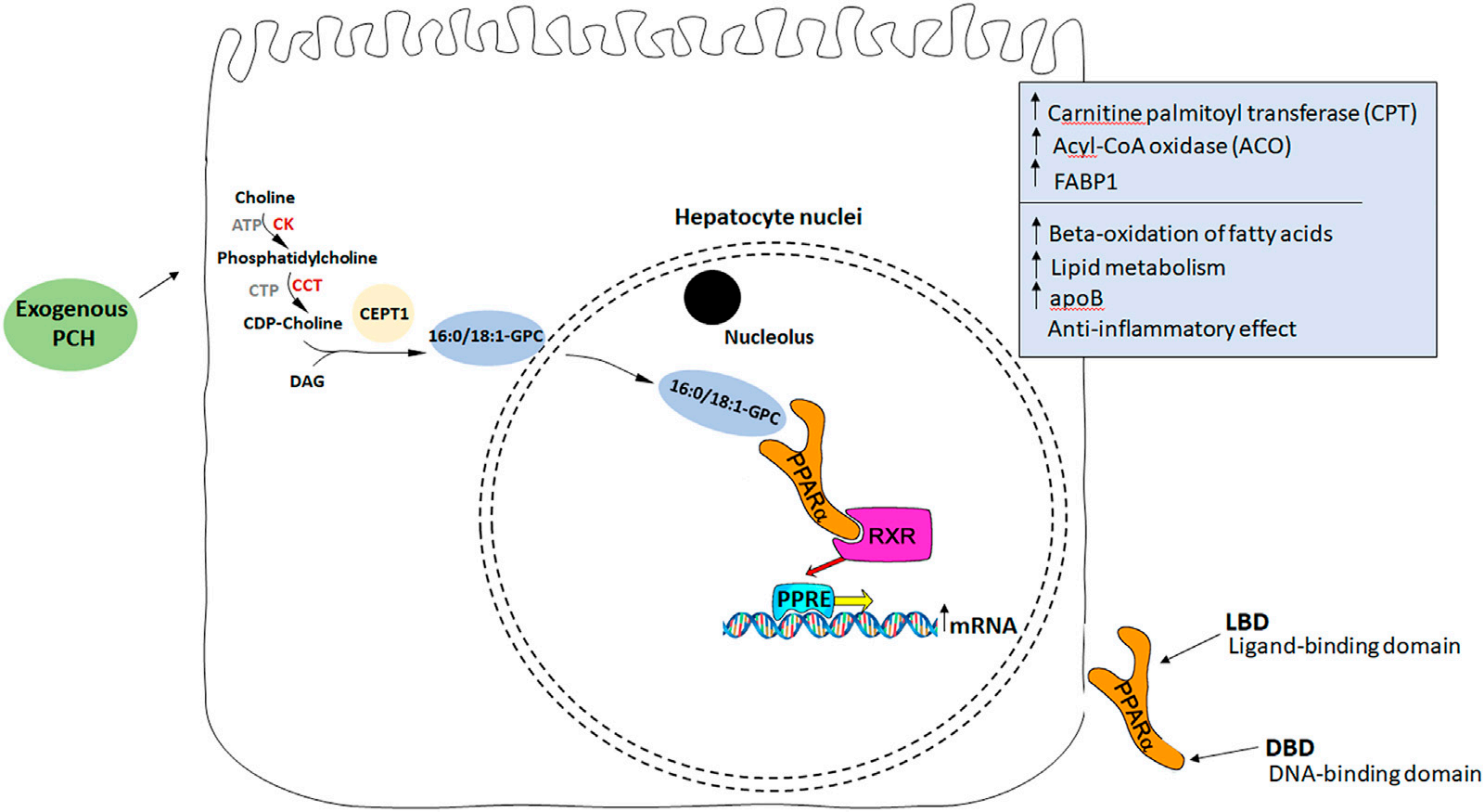
PCH as an endogenous ligand of PPARα. Exogenous PCH means PCH administered with essential phospholipids. (1) PCH stimulates endogenous synthesis of 16:0/18:1-GPC binding with PPARα receptors. (2) After PPARα activation it binds to another transcription factor, RXR (retinoid-x receptor). (3) The formation of heterodimeric complexes leads to their transport into the nucleus. (4) Heterodimeric complexes bind to the specific sequence, peroxisome proliferator response element (PPRE), localized in the promoters of different genes responsible for ACO, CPT, and FABP1 synthesis. (5) *Acyl-CoA oxidase participates in the oxidation of fatty acids. *Carnitine palmitoyltransferase is responsible for the transfer of FA from the outer to the inner membrane for oxidation of the fatty acid inside the mitochondria. *FABP1 provides an appropriate VLDLs assembly.

Considering PPAR and fatty liver, Montagner et al., 2016 showed that hepatocyte PPARα deletion in mice impaired fatty acid catabolism leading to hepatic lipid accumulation even in a fasting state in two steatosis models (Montagner et al., 2016). On the other hand, choline deficiency led to reduced PPARα expression and consequently to reduced expression of PPARα-dependent enzymes: ADRP, DGAT2, CPT1a, and FABP4 (Csak et al., 2015). These enzymes are known to be responsible for fatty acid metabolism, VLDL storage, synthesis, and secretion. PPARα also regulates beta-oxidation of fatty acids influencing CPT transcription (Mello et al., 2016). In turn, the PCH diet in the fatty liver model led to an increase in CPT activity in rats (Buang et al., 2005). With that in mind, and knowing that PCH may be a potential endogenous ligand for PPARα (Lamaziere and Wolf, 2010), it is considered a target molecule being affected directly by PCH (Figure 2).

The following studies provided additional data supporting this pathway hypothesis. It was shown that an endogenously synthesized phospholipid can bound to PPARα isolated from a mouse liver and activate it. Such binding increased under conditions that induce FAS activity and was displaced by systemic injection of a PPARα agonist. Mass spectrometry identified the species as 1-palmitoyl-2-oleoyl-sn-glycero-3-phosphocholine (16:0/18:1-GPC). Interactions of 16:0/18:1-GPC with the PPARα ligand-binding domain and co-activator peptide motifs were comparable to those of PPARα agonists. Portal vein infusion of 16:0/18:1-GPC induced PPARα-dependent gene expression and decreased hepatic steatosis (Chakravarthy et al., 2009). So, replenishing choline deficiency in the fatty liver with EPLs may stimulate endogenous ligand synthesis to activate PPARα and, therefore, beta-oxidation of fatty acids in the liver leading to steatosis regression.

Another interesting observation was made when PCH treatment of myotubes was analyzed. It increased FA uptake and fatty acid-binding protein 3 (FABP3) expression. Remarkably, the effect of PCH on promoting FA utilization in muscles was abolished in PPARα-null mice and PPARα-depleted myotubes (Wang et al., 2020).

Thus, improved blood lipid profile in NAFLD patients treated with EPLs may be explained with the effect of PCH not only on the liver PPARα but also on the muscles PPARα. We believe that this pharmacodynamic potential requires separate research.

## Liponeogenesis Inhibition

Considering PPAR-δ/γ types, we failed to find consistent data showing that PCH may be an effective agonist of these PPAR subtypes. This is consistent with the data from Chakravarthy et al. (2009). At the same time, it should be mentioned that PCH is not the only molecule potentially influencing PPARα. The PPAR family is currently one of the key target molecules in terms of NAFLD treatment. For instance, molecules such as lanifibranor and elafibranor are pan-PPAR and PPARα/δ agonists, respectively. However, a phase III study of elafibranor (RESOLVE-IT, NCT02704403) was terminated due to lack of efficacy, while lanifibranor showed promising results in phase IIb (Francque et al., 2021). There is currently an ongoing phase II lanifibranor study evaluating its effect on liver steatosis (NCT03459079). The results are expected in 2022.

Considering the complexity of PPAR regulation, another promising pathway should be mentioned here, influencing PPAR not directly but via sirtuins: a group of proteins of the silent information regulator two family. Sirtuins are class III histone deacetylases, which is implicated in many cellular and physiological functions, including hepatic glucose and fatty acid metabolism, mitochondrial function, hepatic gluconeogenesis, insulin secretion, and maturation of fat cells. Seven mammalian sirtuins (SIRT1–SIRT7) have been identified and shown to share the same conserved NAD binding site and catalytic core domain, but with different N and C termini (Nogueiras et al., 2012). SIRT1, 6, and 7 are localized mainly in the nucleus while SIRT3, 4, and 5 are localized in the mitochondrial matrix, and SIRT2 is predominantly cytoplasmic. Decreased expression of SIRT1, SIRT3, SIRT5, and SIRT6 and increased expression of SIRT4 in NAFLD patients compared to the control group was demonstrated. This was associated with increased expression of lipogenic genes including sterol regulatory element-binding protein-1, fatty acid synthase, and acetyl-CoA carboxylase (Wu et al., 2014).

Considering PPAR, it is worth mentioning that SIRT4 modulates the activity of various target substrates involved in fatty acid metabolism and, in particular, suppresses PPAR-α (and beta-oxidation), while SIRT1 and SIRT3 induce fat utilization (Han et al., 2019). SIRT4 also may decrease the amino acid-stimulated insulin secretion by inhibiting the glutamate dehydrogenase activity in pancreatic β-cells (Tarantino et al., 2014). Interestingly, data are suggesting that physical exercises may change the intracellular NAD+/NADH ratio and, therefore, alter the activity of some NAD + -dependent sirtuins. Considering pharmacotherapeutic agents, current data do not allow us to point at effective agonists/antagonists with the effect supported by clinical findings (Nassir and Ibdah, 2016). This is a new direction of research requiring a better understanding of sirtuin functions, targets, and regulation (Elkhwanky and Hakkola, 2018). We would like to draw the attention of the readers to some publications in this field, while not intending to cover it comprehensively since it is not the main goal of this review (Xu et al., 2010; Ding et al., 2017).

### SREBP-dependent Lipid Accumulation Through the GPAT Activation

Triacylglycerol (TAG) is synthesized in most human cell types through the glycerol phosphate pathway. The first step in this process is the acylation of glycerol-3-phosphate by glycerol-3-phosphate acyltransferase (GPAT). Subsequent steps include fatty acid translocation to lysophosphatidic acid (LPA) by AGPAT (1-acylglycerol-3-phosphate-O-acyltransferase also known as LPA acyltransferase) to form a phosphatide and then diacylglycerol (DAG). The final conversion of DAG into TAG is catalyzed by diacylglycerol acyltransferase (DGAT) (Samuel et al., 2004; Petersen et al., 2007; Liu et al., 2010).

Lipogenesis enzymes regulation is carried out by specific transcription factors. For example, the GPAT enzyme, which plays a primary role in the initiation of TAG synthesis, is regulated at both transcriptional and post-transcriptional levels. Researchers found a 20-fold increase of GPAT1 mRNA in mice liver when resuming a high-carbohydrate diet after fasting (insulin-related stimulation), which was associated with liver lipogenesis activation (Coleman and Lee, 2004). At the same time, SREBP-1c (sterol regulatory element-binding protein 1c) is considered a key transcriptional activation factor of GPAT1. Shown below is how PCH and its membrane concentration may influence SREBP activation and, therefore, GPAT expression and lipogenesis.

### SREBPs as a Target for PCH

SREBPs (sterol regulatory element-binding proteins) are transcription factors modulating lipid metabolism (Brown and Goldstein, 1997). SREBP-1a and -1c isoforms preferentially regulate genes responsible for the biosynthesis of fatty acids, phospholipids, and TAG, whereas SREBP-2 controls cholesterol metabolism (van der Veen et al., 2017). SREBPs are regulated by digested nutrients: SREBP-2 active form depends on cholesterol levels in the endoplasmic reticulum (Horton et al., 2002), whereas SREBP-1 processing is regulated by the insulin level (Browning and Horton, 2004). Activation of the SREBP-1c isoform leads to the synthesis of enzymes involved in lipogenesis. SREBP-1c overexpression causes FA synthesis, a fourfold increase in fatty acid synthase (FAS) expression, and a 10-fold increase in mtGPAT expression (Shimano et al., 1997; Horton et al., 2002). Thus, SREBPs activate TAG synthesis through the regulation of the GPAT enzyme.

Of note, phospholipids also play an important role in the SREBP-1 regulation of lipogenesis (Dobrosotskaya et al., 2002; Seegmiller et al., 2002; Lim et al., 2011; Walker et al., 2011). Walker et al. found in 2011 that phosphatidylcholine synthesis blocked in *C. elegans*, mouse liver, and human cells led to increased SREBP-1-dependent enzymes transcription and lipid droplet accumulation. In nematode *C. elegans*, reduction of PCH synthesis (via CDP-choline or PEMT pathway inhibition) caused SBP-1 (*C. elegans* ortholog of SREBP) processing and folding enhancement. Increased SREBP-1 processing and enhanced lipogenic genes expression were observed in a mouse model with cytidylyltransferase-α (CTα) deficiency and reduced PCH. Thus, we can conclude that SREBP-1 activity depends on PCH level (Walker et al., 2011). Since PCH deficiency models are widely used to induce liver steatosis, this is one of the possible molecular pathways of its development (Vance et al., 2007; Zeisel, 2008).

**FIGURE 3:**
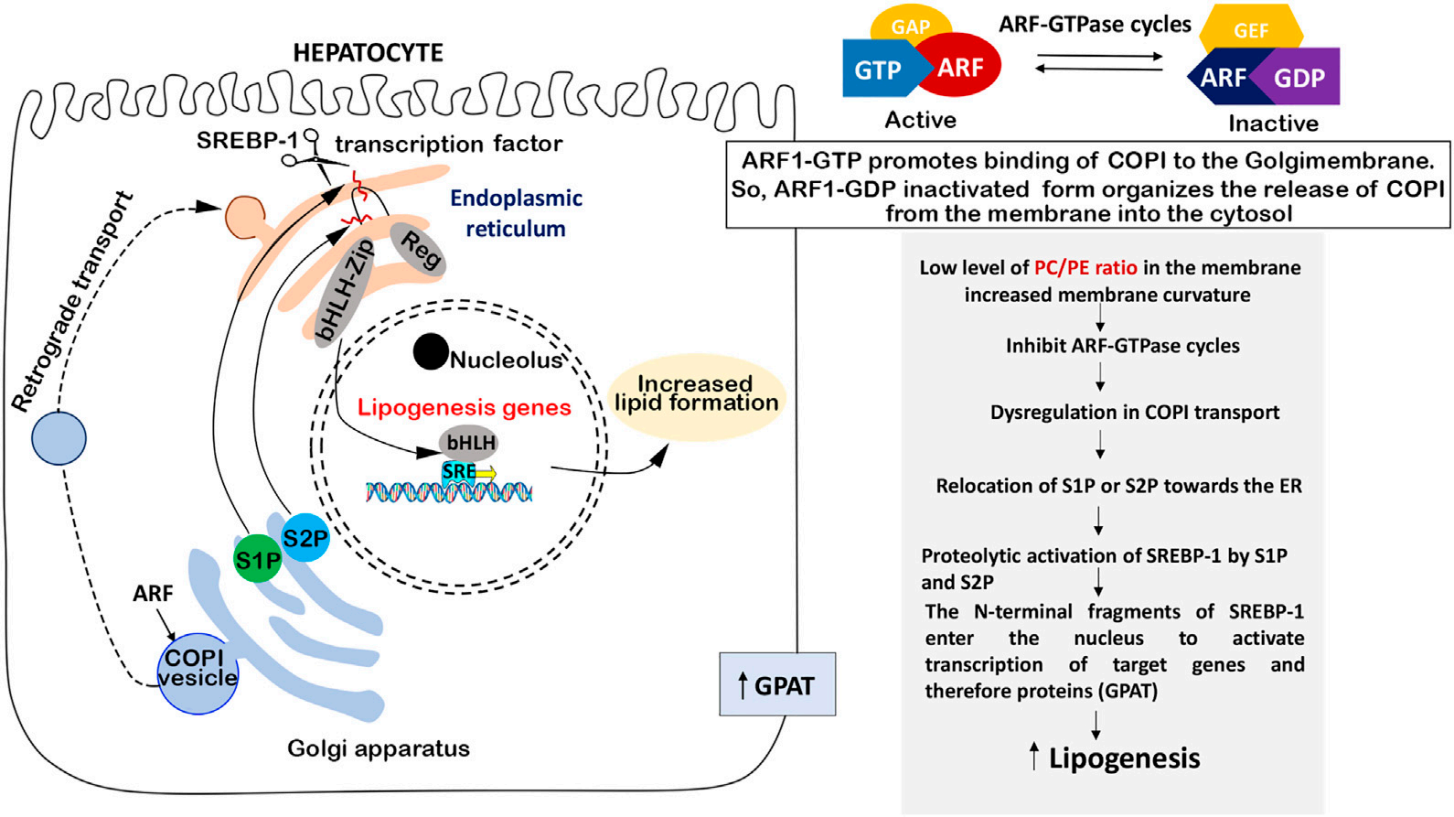
PCH-dependent SREBP regulation**.** PCH enrichment in membranes decreases the membrane curvature which leads to ARF-GTFase cycle activation and COPI transport repair without excess relocation of S1P and S2P proteinase from Golgi to the ER. This means inhibition of SREBP-1 and, therefore, SREBP-1-dependent lipogenic enzymes.

The main component of cell membranes is PCH. SREBP-1 activation occurs within the endoplasmic reticulum (ER) and Golgi membranes. It was hypothesized that changes in these membranes could lead to increased SREBP-1 activity (Walker et al., 2011). The PCH/PE ratio is responsible for membrane fluidity and curvature and plays a fundamental role in the regulation of cell metabolism, so depletion of PCH can alter protein transport and lipid accumulation and can be even associated with NAFLD in humans (Testerink et al., 2009; Walker et al., 2011). Thus, changes in PCH levels may alter membrane function leading to SREBP-1 activation (Walker et al., 2011). PCH level can affect the localization of serine protease 1 and 2 (S1P, S2P) localized in the Golgi membrane and necessary to convert the SREBP-1 precursor into an active nuclear transcription factor (Figure 3).

S1P and S2P in Golgi membranes are necessary for SREBP-1 maturing, and its transformation takes place in the ER, so S1P and S2P should be transferred from the Golgi membrane to the ER to activate SREBP-1. S1P and S2P translocation is regulated by COPI-coated transport vesicles, initiated with ADP-ribosylation factor (ARF1). Interestingly, ARF1 is a small GTPase of the Ras superfamily (Kahn and Gilman, 1984) and it may be suppressed by ARF-GTPase repressor (ARF-GAP) in case of the membrane curvature increase caused by a low level of PCH in the membrane (Dobrosotskaya et al., 2002; Seegmiller et al., 2002). In several models, it was shown that blocked ARF1 led to active SREB-1 nuclear accumulation. So, active SREBP-1 nuclear accumulation takes place in the case of low PCH level through the following mechanism: low PCH level increases the membrane curvature which may affect ARF signaling, deregulate COPI transport, and shift the distribution of S1P or S2P toward the ER, where they cleave and activate SREBP-1 (Walker et al., 2011). Another important thing is that the PCH-mediated SREBP activation mechanism is not affected by SREBP-2 activation (Walker et al., 2011). This means that PCH does not interfere with cholesterol metabolism and does not block its metabolic pathways.

Considering all the above, we can conclude that replenishing PCH deficiency can normalize the PCH/PE ratio and SREBP-1 activity and, therefore, suppress the synthesis of fatty acids and prevent the accumulation of fat in the liver.

## Fatty Acids and TG Secretion/Evacuation

**FIGURE 4:**
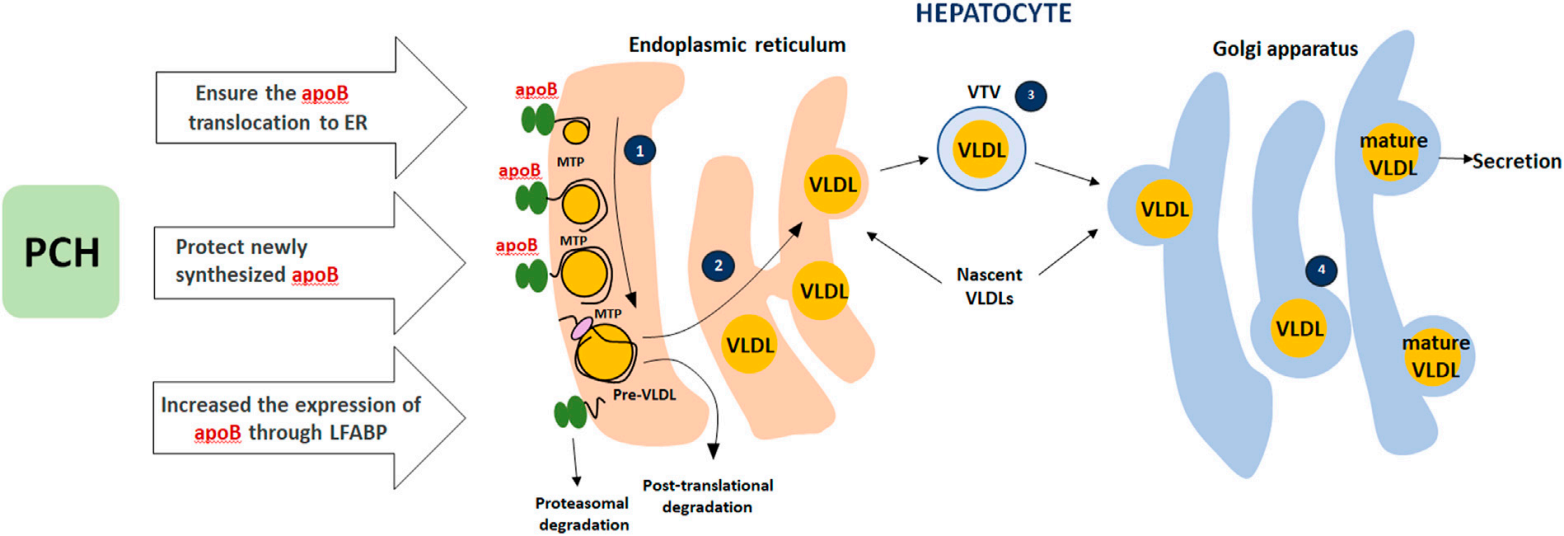
Role of PCH in VLDLs secretion**.** (1) The assembly of VLDLs starts when apoB100 is translocated to the lumen of the endoplasmic reticulum (ER) and interacts with microsomal triglyceride transfer protein (MTP); *apoB100 that does not interact with MTP goes through proteasomal degradation. (2) MTP-dependent lipidation associated with pre-VLDL formation (pre-VLDL that is not converted to a mature lipoprotein will go through post-translational degradation). (3) Transport of nascent VLDLs to Golgi carried out using a specialized transport vesicle: the VLDL transport vesicle (VTV). (4) Then nascent VLDLs undergo a number of essential modifications on the Golgi lumen (further VLDL lipidation). PCH plays a key role in apoB translocation to the ER, protects newly synthesized apoB, and increases apoB expression through LFABP.

PCH appears to ensure the translocation of apoB (apolipoprotein B) from the cytosol to the lumen of the endoplasmic reticulum. This part of the modification is crucial in the early stages of VLDL assembly. Appropriate choline level protects newly synthesized apoB from intracellular degradation during the migration of apoB from the ER to the Golgi apparatus. PCH as an appropriate PPARα ligand increases the expression and biosynthesis of liver fatty acid-binding protein (LFABP). High expression of FABP1 isoform increases the expression of apoB-100, thus ensuring correct assembly of VLDL in the ER lumen (Olofsson and Borén, 2012; Tiwari and Siddiqi, 2012). This PCH mode of action was not considered the leading one since EPLs lead to steatosis regression in the liver and blood lipid profile improvement with TG, total cholesterol, VLDL, and LDL decrease and HDL increase both in animal models and clinical studies (Gonciarz et al., 1988; Li et al., 2000; Yin, 2000; Wu, 2009; Sas et al., 2013; Maev et al., 2020a, Maev et al., 2020b; Popovic and Dajani, 2020). At the same time, taking into account the PPAR section of our paper, this effect matches PPARα activation in muscles and fatty acids uptake. These may work together and contribute to the fatty acids/TG-enriched lipoproteins elimination from the blood and subsequent utilization in tissues (PPARs as metabolic regulators in the liver: Lessons from liver-specific PPAR-null mice). The potential mechanism is presented in Figure 4.

## Digestion of Dietary Fatty Acids, TG, and Cholesterol

Rampone et al. showed >50% suppression of cholesterol intestinal digestion when incubating it with lecithin in everted rat gut sacs (Rampone, 1972). Several other studies also add to our understanding of the potential PCH effect on fatty molecules digestion. For instance, it was shown that egg sphingomyelin dose-dependently reduced lymphatic cholesterol concentration in rats (Noh and Koo, 2003; Noh and Koo, 2004). When given orally, it significantly reduced plasma triglyceride and cholesterol levels in mice fed a Western-type diet (Duivenvoorden et al., 2006). Surprisingly, in a study by Noh et al. this effect was more prominent with saturated fatty acids in the PCH tail. Several studies in humans showed a similar effect of phospholipids on cholesterol digestion suppression (Beil and Grundy, 1980; Greten et al., 1980; Kesaniemi and Grundy, 1986). Existing data on the inhibitory effect of phospholipids on cholesterol absorption are well summarized elsewhere (Cohn et al., 2010). However, to reproduce this mechanism of action in clinical practice, a huge dose of PCH (at least 10 g daily) seems to be required. Possible mechanisms for the inhibition of cholesterol absorption by phospholipids have already been presented by Cohn et al. (2008):

1) Excess PCH interferes with efficient micellar PL hydrolysis: a prerequisite for mucosal uptake of cholesterol.
2) PCH surplus alters the physicochemical properties of mixed micelles (i.e., their size, composition, and/or biological characteristics) resulting in reduced absorption of cholesterol.
3) PCH affects the membrane characteristics of enterocytes or has a direct effect on cellular cholesterol transporters that regulate intestinal cholesterol uptake.

## Conclusion

Four possible mechanisms of PCH-induced steatosis regression showed both *in vivo* (Buang et al., 2005; Lee et al., 2014) and in clinical studies (Gundermann et al., 2016; Maev et al., 2020b; Popovic and Dajani, 2020) are discussed and the following are considered as relevant:

1) Stimulation of fatty acids beta-oxidation in hepatocytes (through PPARα and PPAR-dependent enzymes: acyl-CoA oxidase and carnitine palmitoyltransferase)
2) Liponeogenesis inhibition in hepatocytes (through SRBEP-1 and SRBEP-dependent enzymes, mainly glycerol-3-phosphate acyltransferase)
3) Fatty acids evacuation followed by their uptake and utilization in muscles (through the role of PCH both in VLDL formation and evacuation and the PPARα activation in the muscles)

Of course, this concept requires further *in vitro* and *in vivo* testing to obtain a true picture of the PCH mode of action in clinical practice. We believe that these data contribute to a better understanding of the clinical effect of EPLs and may help design further studies in this field. It is even more important considering the new MAFLD concept and steatosis as a universal phenotypical sign of metabolic disorder in the liver that should be diagnosed and treated.
